# Thalidomide attenuates nitric oxide mediated angiogenesis by blocking migration of endothelial cells

**DOI:** 10.1186/1471-2121-7-17

**Published:** 2006-04-04

**Authors:** KP Tamilarasan, Gopi Krishna Kolluru, Megha Rajaram, M Indhumathy, R Saranya, Suvro Chatterjee

**Affiliations:** 1 Vascular Biology Lab, AU-KBC Research Centre, Anna University, Chennai, India; 2 Worked as summer students in June-July 2005. They are B.Tech students from the Vivekanandhaa College of Engineering for Women, Tiruchengode, Namakkal, TN, India

## Abstract

**Background:**

Thalidomide is an immunomodulatory agent, which arrests angiogenesis. The mechanism of anti-angiogenic activity of thalidomide is not fully understood. As nitric oxide is involved in angiogenesis, we speculate a cross-talk between thalidomide and nitric oxide signaling pathway to define angiogenesis. The aim of present study is to understand the mechanistic aspects of thalidomide-mediated attenuation of angiogenesis induced by nitric oxide at the cellular level.

**Methods:**

To study the cellular mechanism of thalidomide-mediated blocking of angiogenesis triggered by nitric oxide, we used two endothelial cell based models: 1) wound healing and 2) tube formation using ECV 304, an endothelial cell line. These cell-based models reflect pro-angiogenic events *in vivo*. We also studied the effects of thalidomide on nitric oxide mediated egg yolk angiogenesis. Thalidomide could block the formation of blood vessels both in absence and presence of nitric oxide. Thalidomide effects on migration of, and actin polymerization in, ECV 304 cells were studied at the single cell level using live cell imaging techniques and probes to detect nitric oxide.

**Results:**

Results demonstrate that thalidomide blocks nitric oxide-mediated angiogenesis in egg yolk model and also reduces the number of tubes formed in endothelial cell monolayers. We also observed that thalidomide arrests wound healing in presence and absence of nitric oxide in a dose-dependent fashion. Additionally, thalidomide promotes actin polymerization and antagonizes the formation of membrane extensions triggered by nitric oxide in endothelial cells. Experiments targeting single tube structure with thalidomide, followed by nitric oxide treatment, show that the tube structures are insensitive to thalidomide and nitric oxide. These observations suggest that thalidomide interferes with nitric oxide-induced migration of endothelial cells at the initial phase of angiogenesis before cells co-ordinate themselves to form organized tubes in endothelial cells and thereby inhibits angiogenesis.

**Conclusion:**

Thalidomide exerts inhibitory effects on nitric oxide-mediated angiogenesis by altering sub-cellular actin polymerization pattern, which leads to inhibition of endothelial cell migration.

## Background

Thalidomide, α-(N-phthalimido) glutarimide is an immunomodulatory agent, which is used as a drug to treat multiple myeloma and other types of cancers [1]. The drug thalidomide, first synthesized in 1954 [2,3], was widely prescribed to treat morning sickness in pregnant women in the early 60s. However, thalidomide became anathema when it was found to be seriously teratogenic having caused serious birth defects in more than 10,000 newborns. It was subsequently banned in Europe. As scientists probed further into the causes for teratogenicity, they realized the innate potency of this drug to treat other diseases [4]. Now, a quarter of a century later, it appears that it may be a miracle drug for such diseases as cancer, AIDS and SLE. In August 1998, Food and Drug Administration (FDA) approved Thalidomide for sale in the USA for chronic treatment of erythema nodosum leprosum (ENL), a painful inflammatory dermatological reaction of lepromatous leprosy [4].

Thalidomide has anti-angiogenic properties that are independent of its immunomodulatory effects [5,6]. Its involvement in angiogenesis is being studied extensively as angiogenesis is one of the most important developments in metastazing tumors. Thalidomide has been shown to inhibit the expression of vascular endothelial growth factor (VEGF) and IL-6, which is believed to be the mechanism for the attenuation of angiogenesis by thalidomide [7]

Nitric oxide (NO), produced by the enzyme nitric oxide synthase (NOS), an important second messenger in many signaling pathways, is a potent vasodilator [8]. NO is produced by the action of NOS in the transformation of arginine to citrulline and activates soluble guanylate cyclase to produce cGMP [9]. NO is involved in angiogenesis and endothelial cells (EC) migration [10]. Recent publications suggest that endothelial-derived NO is required for Ang1-induced angiogenesis and that the PI3-kinase signaling mediates the activation of eNOS and NO release in response to Ang1 [11]. NO is also instrumental in promoting Ang1-induced angiogenesis in combination with HSP90 and Akt in coronary artery endothelium [12].

As thalidomide interferes with angiogenesis, a process in which NO also plays a crucial role, we speculate a cross talk between thalidomide and NO signaling pathway. Currently, the involvement and interaction of thalidomide and the NO signaling pathway is not known. The mechanistic aspects of thalidomide action need to be elucidated further. The aim of present work was to study the mechanistic aspects of the attenuation of NO-driven angiogenesis due to thalidomide at the cellular level. Our study indicates that thalidomide attenuates NO-driven angiogenesis by blocking migration of ECs even before any tube structure had been formed, which further hints an interaction between thalidomide and NO signaling.

## Methods

### Materials

Dulbecco's modified Eagle's medium (DMEM), from Hi-Media, Mumbai, India. Fetal bovine serum (FBS) was from Invitrogen Life technologies (Gaithersburg, MD). Thalidomide was purchased from Sigma Chemical Co (St. Louis, MO), and phalloidin Alexa Fluor 568 (phalloidin) from Molecular Probes (Portland, OR, USA). All other chemicals were at least of the reagent grade.

### Cell culture

Human umbilical vein endothelial cells (ECV 304) were cultured in DMEM supplemented with 10 % FBS (v/v) and 1% penicillin (w/v) and streptomycin (w/v).

### Endothelial tube formation assay

ECV 304 cells were seeded on collagen (collagen type I) plated 12-well plates with 60% cell density. After 7 hours of incubation, 500 μmol of sodium nitroprusside (SNP), a NO donor, was added to the cells. After another 17 hour period, cells were treated with thalidomide at different concentrations (50, 100 and 150 μg/ml) and incubated for 8 hours. All the incubations were performed in an incubator at 37°C and under 5% CO_2 _95% air. The number of tubes was counted under bright field phase contrast microscope. Only the complete ring structures created by 3 to 5 ECs were counted as tubes.

### Single cell migration assay

Cell migration was assessed by a wound healing method [10]. One million ECV 304 cells in 2 ml of DMEM/10% FBS were seeded in a 35 mm dish. Twenty-four hours later, when the cells reached confluence, a linear wound was made by scratching the monolayer with a 1 mm wide sterile plastic scraper. As per the experimental protocol described elsewhere [10], cells were washed with PBS, treated with SNP (500 μmol) and incubated for a fixed time period (8 hours unless otherwise stated) with and without thalidomide at different concentrations. Light microscopy images were taken with 10× and 40× magnifications.

### Boyden's chamber based migration assay

Trypsinized ECV 304 cells were used for migration assay using Boyden's chamber, which is a two-chamber system. The upper and lower chambers are separated by a collagen coated 8-μm pore size polycarbonate membranes. ECV 304 cells were loaded in the upper well with thalidomide alone or thalidomide plus SNP. Lower well was filled with DMEM. The chambers were then incubated at 37°C, 5% CO_2 _for 3 hours. Cells were migrated across the membrane and stuck to the lower part of the membrane. After the incubation, the polycarbonate membrane was fixed and stained with propidium iodide, a fluorescent nuclear probe. Endothelial cell migration activity was quantified as the number of migrated cells on the lower surface of the membrane. Cell number was counted before and after experiments to quantify the proliferation status of the loaded cells in Boyden's chamber.

### Egg yolk angiogenesis assay

Fourth day incubated eggs were collected from the Poultry Research Station, Nandanam, Chennai. Eggs were broken and gently plated on a cellophane bed in Petri dishes under sterile conditions. Egg yolks were incubated with 500 μmol SNP on a filter paper disc for 6 hours. Thalidomide discs (25, 50,75 μg/ml) were then placed on the egg yolks and were incubated for another 6 hours. Images were taken using a Kodak digital camera at 0, 6 and 12 hours of incubation. Quantification of angiogenesis was performed by using Scion Image, Release Alpha 4.0 3.2 and Adobe Photoshop version 6.0.

### Fluorescence microscopy

ECV 304 cells were cultured on cover glasses in 12-well plates till they reach 40% confluency before starting the experiments. ECV 304 cells were incubated with 500 μmol SNP for 15 minutes. Next, thalidomide was added at different concentrations (25, 50, 75 μg/ml) to the cells. After 15 minutes of incubation at 37°C, the cover slips were washed gently with PBS and cells were fixed in 2% paraformaldehyde for 7 minutes, permeabilized with 0.1% Triton X -100 for 2 minutes and incubated with phalloidin alexa fluor 568 (phalloidin) (0.5 μM final concentration) for 1 hour. The fluorescence of phalloidin bound to F-actin was viewed under NIKON TE2000-U fluorescent microscope at 560 nm emission. Photographs were taken with an Andor CCD camera attached to the microscope.

### Statistical analysis

Data is presented as mean + SE. Data was analyzed using paired and unpaired Student's t-tests as appropriate. A *P *value smaller or equal to 0.05 was selected as a criterion for a statistically significant difference.

## Results

### Thalidomide blocks angiogenesis

**Figure 1:**
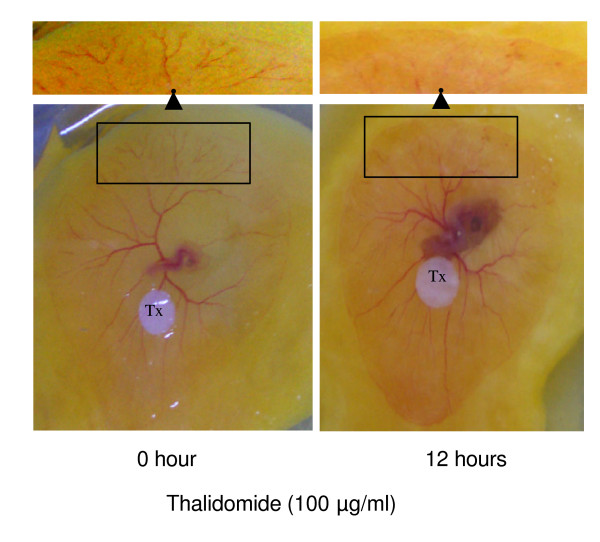
**Thalidomide blocks angiogenesis**. Fourth day incubated chicken eggs were broken, entire egg contents were transferred into a sterile petri dish. In vascular bed, heart and blood capillary are visible on 0 hour panel (left). Next, the vascular bed was incubated with 100 μg/ml thalidomide disc, placed on the vascular bed for 12 hours. It is evident from the representative images that 100 μg/ml thalidomide retarded the growth of the blood capillaries at the edges of vascular bed. A 70% loss of blood vessels (inset on top of the panels) was estimated by counting the number of red pixels using an analytical module of Adobe Photoshop ver. 6.0.

An egg yolk vascular bed model is an angiogenesis model in which one can track and observe the development of cardiovascular system including angiogenesis [13]. This model was chosen for our experiments to test the effects of thalidomide on angiogenesis. Yolks from the 4^th ^day-fertilized eggs with semi-developed vascular bed were plated on petri dishes and treated with thalidomide (50, 100, 150 μg/ml) for 12 hours. Egg yolk vascular beds treated with 150 μg/ml thalidomide showed necrotic effects on vascular bed (images not shown) while vascular beds treated with 50 and 100 μg/ml of Thalidomide were healthier. Capillary growth was significantly inhibited by thalidomide without disturbing other vasculature (Figure 1). A 12-hour treatment with thalidomide (100 μg/ml) caused a 70% loss in terminal capillaries in the egg yolk vascular bed (Figure 1).

### Thalidomide attenuates tube formation in EC monolayer

**Figure 2:**
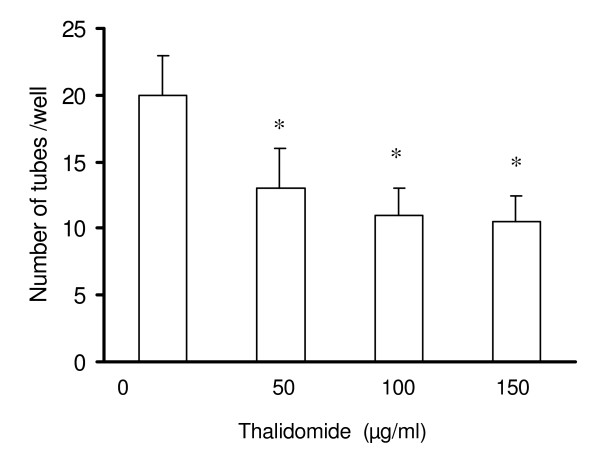
**Thalidomide attenuates tube formation in ECs**. Trypsinized ECs in suspension culture were seeded on collagen coated 12 well plates with 70%cell density. After 24 hours, the ECs were treated with different concentrations of thalidomide, (50, 100 and 150 μg/ml) and incubated at 37°C/5%CO_2 _for 12 hours and thereafter, total number of tubes were counted. Number of tubes was reduced in all, 50 100 and 150μg/ml thalidomide treated ECs respectively.

To explore the effects of thalidomide on endothelial functions, we used two cell-based models, tube formation and wound healing, in monolayer cultures of immortalized ECV 304 cells. ECs have natural tendency to organize themselves to form three-dimensional structures (tube formation) in monolayer culture [14]. We checked the effects of thalidomide on tube formation after 12 hours of incubation and observed a dose-dependent reduction in the number of tubes formed (Figure 2).

### Thalidomide arrests wound healing in EC monolayer

**Figure 3:**
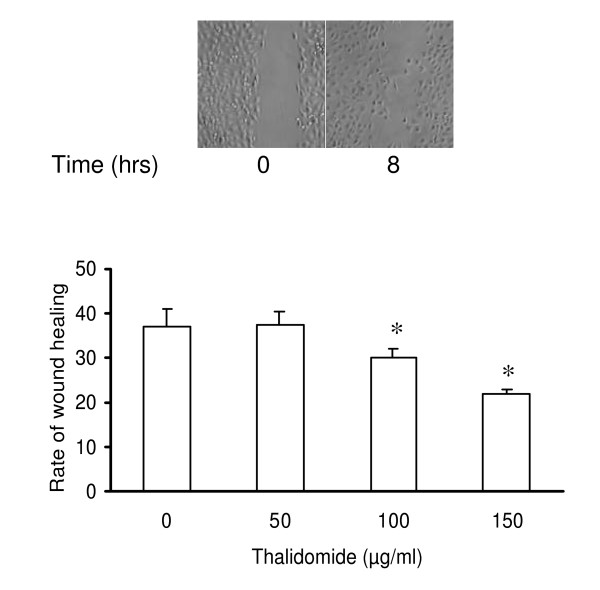
**Thalidomide impairs basal wound healing in EC monolayer**. Wound was created with a sterile plastic scraper and images were taken at 0 and 8 hours. A representative image from top panel shows how the proliferation of ECs heal the wound in 8 hours. The rate of wound healing was quantified (in the graph below) from the images using Scion Image, Release Alpha 4.0 3.2 and Adobe Photoshop version 6.0. Analysis of the data depicts that the rate of wound healing is slower in 100 and 150 μg/ml thalidomide treated cells. The asterisks represent the level of significance. * *P *< 0.05 compared to control.

**Figure 4:**
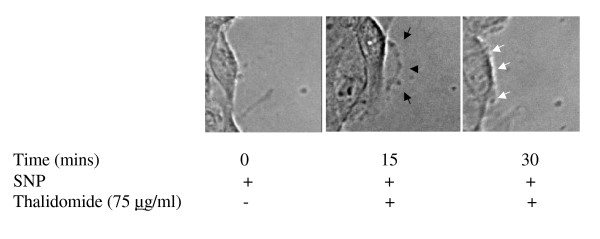
**Thalidomide attenuates NO mediated migration in ECs**: After the wound was created in confluent ECs, they were treated with 500μM SNP (NO donor) and incubated for 15 minutes at 37°C/5% CO_2_. Next, the cells were treated with 75 μg/ml thalidomide and incubated for another 15 minutes. Images of the wound-edge were taken at 0 minutes, 15 minutes and 30 minutes with a 40× magnification objective lens mounted on Nikon TE2000-U inverted microscope. Arrows indicate the growing phases of the wounds after 15 minutes of incubation with SNP. Arrows in the middle panel indicates the growing phase of ECs under thalidomide treatment, while thalidomide arrests the SNP mediated migration of the cells after 15 minutes of thalidomide treatments (white arrows in the right panel).

The wound healing model is used to estimate the migration potential of the ECs in monolayer culture [15]. Wounds were artificially made in EC monolayer before addition of thalidomide (50, 100 and 150 μg/ml) and incubation for another 8 hours (Figure 3). Thalidomide caused 20 % and 10% reduction in the rate of wound healing at 150 and 100 μg/ml respectively but had no effect at the lowest concentration (50 μg/ml) used (Figure 3). Additionally, a drastic change in morphology, characterized by the rounding up of 30% of total cell population but no loss of viability (data not shown), was observed in cells submitted to thalidomide at 150 μg/ml. Because this was not an expected phenomenon in angiogenesis *in vivo*, lower concentrations (25, 50 and 75 μg/ml) were tested. Further, we performed trypan blue viability assay on thalidomide (25, 50 and 75 μg/ml) treated ECs. Results showed that thalidomide does not interfere with the viability of ECs (data not shown). A simultaneous close capture of the edges of the artificially created wounds was used to obtain information about migration at the single cell level (Figure 4). Thalidomide mostly at 50 (data not shown) and 75 μg/ml, antagonized membrane extensions in ECs and probably thereby reduced the rate of migration of the cells at the wound edges (Figure 4).

### Thalidomide induces cytoskeletal rearrangements in ECs

**Figure 5:**
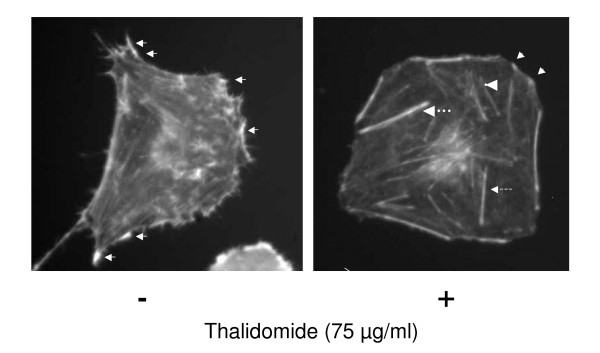
**Cytoskeletal rearrangements by thalidomide**. ECs were treated with different doses of thalidomide (25, 50 and 75 μg/ml). Representativeimage of ECs treated with 75 μg/ml thalidomide, depicts that thalidomide induces central micro filaments (arrows, thalidomide +) and reduces the number of lamellipodia in comparison to the control cell treated with DMSO, the vehicle only (arrows, thalidomide -). Further, we observed three sub populations of the central microfilaments in thalidomide treated cells, thick (thicker broken arrow), medium thick (thin broken arrow) and a thin centre microfilament (arrow head).

Recent evidence suggest that thalidomide is involved in cell migration, development and morphology as observed with the mesenchymal neural crest cells, which are the developmental precursors of the corneal endothelium and stroma [16]. Thalidomide appears to predominantly inhibit leukocyte rolling which suggests that the inhibition of LPS- or muTNF-alpha-induced leukocyte extravasation by thalidomide may account for some of its clinical activities [17]. We treated ECV 304 cells with thalidomide at concentrations (25, 50 and 75 μg/ml) that did not affect cell viability (data not shown) for 30 minutes. Cells were then stained with phalloidin, a water-soluble compound that selectively binds with F-actin at nanomolar concentrations [18]. Thalidomide, at 75 μg/ml, induced the formation of central microfilaments (Figure 5, arrows, thalidomide +) and reduced the number of lamellipodia, in comparison to what was observed in control cells treated with DMSO (solvent for thalidomide) (Figure 5, arrows, thalidomide -). Furthermore, we observed that central microfilaments in thalidomide-treated cells presented a thick, medium thick or a thin centre (Figure 5).

**Figure 6:**
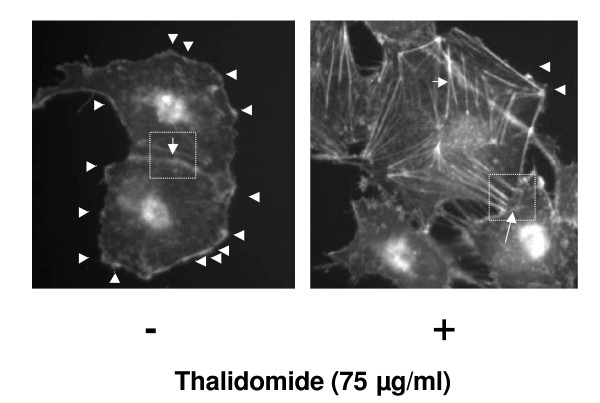
**Thalidomide induced a sharp fall in actin polymerization pattern at the cell-cell interface**. ECs doublets were treated with and without thalidomide respectively, as described elsewhere. A representative image of the ECs treated with 75 μg/ml thalidomide demonstrates a drastic drop in the degree of actin polymerization at the cell-cell interface, and that the arrays of stress fibers are formed under thalidomide challenge in ECs (see boxes in the images, figure 3b).

Next, similar experiments were performed using cell doublets where two ECV 304 cells are attached at the cell-cell interface. The representative image (Figure 6) indicates that a treatment with thalidomide at 75 μg/ml prevented the formation of lamellipodia extensions on the cell surface and induced actin polymerization to form central microfilaments (Figure 6). Cell-cell communication is an essential part for ECs to tune signal transduction to control cellular migration, proliferation and angiogenesis [8]. To understand the effects of thalidomide on actin polymerization at the cell-cell interface we treated EC doublets with thalidomide and observed a drastic drop in the degree of actin polymerization at the cell-cell interface (Figure 6).

### Thalidomide attenuates NO-mediated angiogenesis in egg yolk models and tube formation in EC monolayers

**Figure 7:**
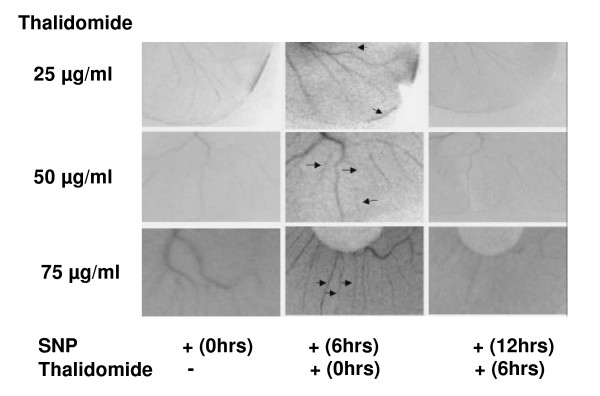
**Thalidomide attenuates NO mediated blood capillaries in invitro egg yolk angiogenesis**. Fourth day incubated chicken eggs were broken, entire egg contents were transferred in sterilized petri dish and their vascular beds were treated with 500 μM SNP and incubated at 37°C/5% CO_2 _for 6 hours. Next, different (25, 50, and 75 μg/ml) concentrations of thalidomide was added to SNP treated vascular beds respectively and incubated for another 6 hours. Images were taken at 0 hours, 6 hours and 12 hours that show that SNP promoted blood vessel formation in the vascular bed in 6 hours (indicated by arrows). All the three concentrations of thalidomide were found to retard SNP mediated growth of blood capillaries at the end of 12 hours incubation.

**Figure 8:**
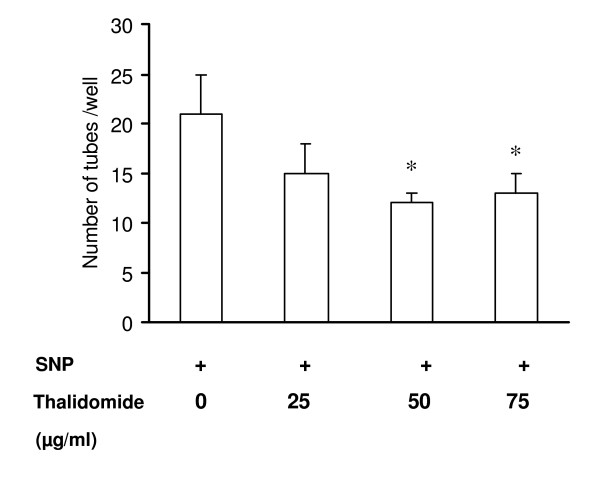
**Thalidomide attenuates tube formation in ECs**. ECs were seeded on collagen coated 12 well plates with 60% cell density, induced with SNP (500 μmol) and incubated at 37°C/5% CO_2 _for 24 hours. A reduction inthe number of NO mediated tubes has been observed by 43% and 40% in 50 and 75 μg/ml thalidomide treated cells respectively.

**Figure 9:**
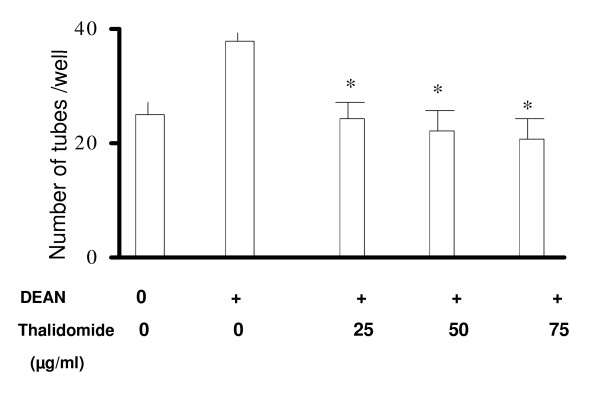
**Thalidomide attenuates DEAN induced tube formation in ECs**. ECs were seeded on collagen plated 12 well plates with 60% cell density, to form monolayers after 24 hours of incubation at 37°C/5% CO_2_. Total number of DEAN (10 μmol) induced tubes were counted after 12 hours of incubation with thalidomide (25, 50, and 75 μgm/ml). * *P *< 0.05 compared to only DEAN treated cells.

**Figure 10:**
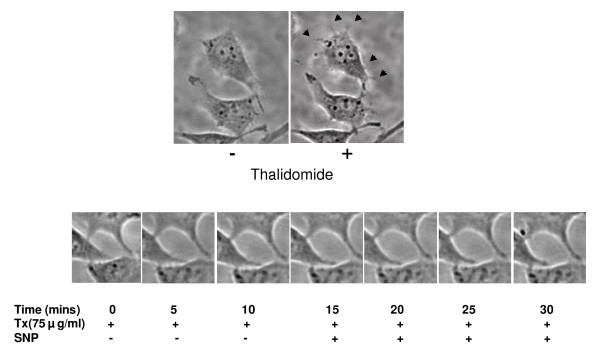
**Time-lapse imaging of thalidomide treated single EC tube structure**. The tube was treated with 500 μmol SNP at 15^th ^minute to find any further effect on the tube structure. No effect of either thalidomide or SNP was observed on the structure and morphology of the tube and the cells as well. However, thalidomide blocked the formation of filopodia, replacing them with broader protrusions on EC surface (arrows in the right panel).

Thalidomide interferes with angiogenesis in egg yolk vascular bed models (Figure 1). Using the same experimental design, egg yolk vascular beds were first treated with SNP for 6 hours to induce angiogenesis and then with thalidomide (25, 50 and 75μg/ml). Representative images (figure 7) are blocks of 0.5 cm × 1 cm. from the whole vascular bed. SNP promoted the formation of new blood vessels and thalidomide arrested NO-mediated blood vessel formation at the growing edges of vasculature even though no dose-dependency could be evidenced (Figure 7). The effect of thalidomide was also tested on NO-mediated tube formation in EC monolayer. The cellular unit of angiogenesis is considered to be the tube structures formed in the EC monolayer [19]. NO-mediated tube formation was reduced by 43% and 40% in cells treated with 50 and 75 μg/ml thalidomide respectively (Figure 8). Thalidomide was also applied to individual tube structures, complete or incomplete, for 15 minutes followed by SNP treatment for another 15 minutes. Cells participating in the tube formation responded to thalidomide challenge and showed membrane activities such as the formation of membrane extensions (Figure 10). Simultaneously, another NO donor, DEA- NONOate (DEAN), was used to induce tube formation in EC monolayer to verify the specificity of NO effects. A concentration of 10 μmol DEAN caused 20% increase in the number of tubes formed in EC monolayer. All three concentrations of thalidomide (25, 50 and 75μg/ml) attenuated DEAN induced tube formation in EC monolayer (Figure 9). However, cells present in complete tubes did not respond to thalidomide or SNP at any concentration. Both the luminal and external sides of the cells present in the tube were smooth and static in response to thalidomide treatments followed by SNP challenge (Figure 10).

### Thalidomide arrests NO mediated wound healing in ECs

**Figure 11:**
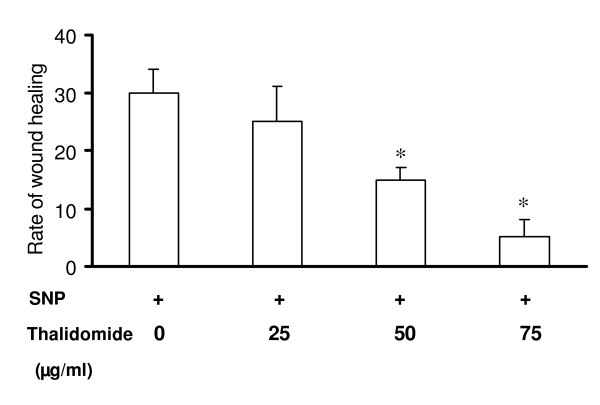
**Thalidomide attenuates NO mediated migration in ECs: **Scratching the EC monolayer with a sterile plastic scraper, a linear wound was made. The cells were incubated with 500 μmol SNP for 1 hour. The SNP treated cells were treated with thalidomide (25, 50, and 75 μg/ml) and incubated for 8 hours. Images were taken at 0 and 8 hours after adding thalidomide to the cells. Quantification of the areas of the wounds was performed by using Scion Image, Release Alpha 4.0 3.2 and Adobe Photoshop version 6.0. The data depicts that rate of SNP mediated wound healing were slower by 50% and 85% in 50 and 75 μg/ml thalidomide treated cells respectively in comparison to that of control. Cells treated with 25 μg/ml thalidomide showed no significant change in SNP mediated wound healing. The asterisks represent the level of significance. * *P *< 0.05 compared to control.

Thalidomide appears to slow down the rate of wound healing in EC (Figure 3). Thalidomide may also modulate NO-mediated wound healing in EC monolayer since this substance attenuated the rate of migration and slowed down the wound healing process triggered with SNP. Thalidomide at 50 and 75 μg/ml slowed down the NO mediated wound healing in ECs by 50% and 80% respectively (Figure 11).

### Thalidomide antagonizes the formation of SNP induced lamellipodia and filopodia

The known anti-angiogenic effects of thalidomide might be attributed to its anti-migratory effects [20,21]. Because no detailed study has been performed so far to understand the anti-migratory effects of thalidomide at the cellular level, we studied the pattern of actin polymerization in ECs treated with SNP and SNP plus thalidomide. SNP induced the formation of filopodia and lamellipodia in EC membrane while subsequent treatments with thalidomide (25, 50 and 75 μg/ml) either inhibited the formation or reduced the number of filopodia and lamellipodia on the EC surface (data not shown).

**Figure 12:**
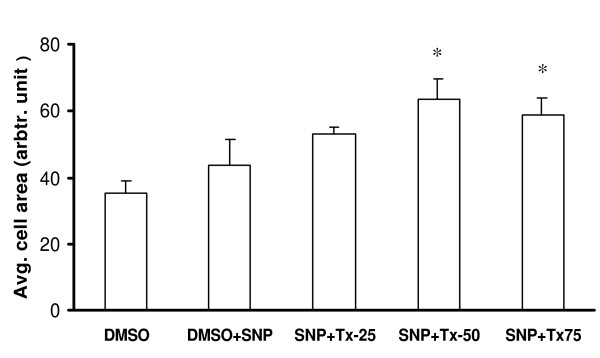
**Thalidomide induced expansion of SNP treated cells**. An increase in average cell area was observed in the cells treated with 50 and 75 μg/ml thalidomide in comparison to that of only SNP treated cells. SNP, Tx25, Tx50 and Tx75 denote 500 μmol SNP, 25, 50 and 75 μg/ml thalidomide respectively. The asterisks represent the level of significance. * *P *< 0.05 compared to control.

**Figure 13:**
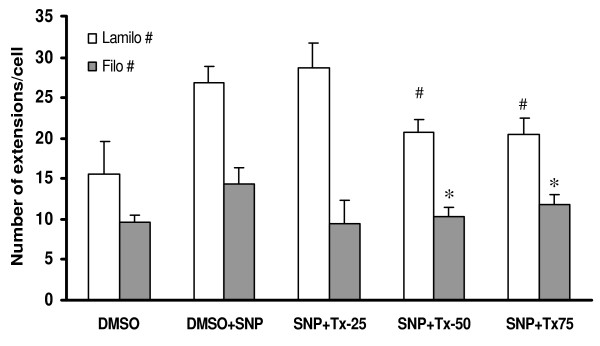
**Thalidomide arrested SNP mediated formation of extensions inECs**. The graph shows that SNP induced formation of lamellipodia and filopodia in comparison to that of DMSO (control) treated cells (data not shown). A further challenge with 25, 50 and 75 μg/ml thalidomide caused a decrease in the number of lamellipodia and filopodia structures in comparison to that of SNP treated cells from 50 and 75 μg/ml groups. SNP, Tx25, Tx50 and Tx75 denote 500 μmol SNP, 25, 50 and 75 μg/ml thalidomide respectively. # and * represents the level of significance for the change of the number of filopodia and lamellipodia respectively when compared that with only SNP groups.

**Figure 14:**
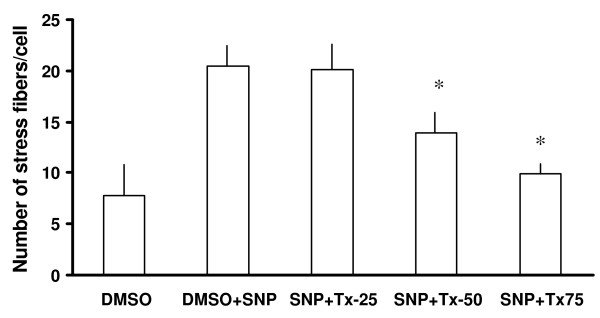
**Thalidomide antagonized the formation SNP induced stress fibers in ECs**. A SNP (500 μmol) treatment induced stress fiber formation in ECs by two and a half fold in comparison to that of DMSO treated (control) cells. When SNP treated cells were challenged with 25, 50 and 75 μg/ml thalidomide respectively, cells from the 50 and 75 μg/ml thalidomide groups showed 30% and 50% drops in the number of stress fibers respectively in comparison to that of only SNP treated cells. SNP, Tx25, Tx50 and Tx75 denote 500 μmol SNP, 25, 50 and 75 μg/ml thalidomide respectively. The asterisks represent the level of significance. * *P *< 0.05 compared to only SNP treated cells.

Further analysis of the images obtained from the experiments described above shows the combination effects of thalidomide and SNP on the migratory pattern of ECV 304 cells. SNP treatments marginally increased the area of the cells, an effect that was accentuated by subsequent addition of thalidomide (25, 50 and 75 μg/ml) (Figure 12). Accordingly a dose-dependent increase in the perimeter of the cells treated with SNP plus thalidomide was recorded (data not shown). Furthermore, the number of lamellipodia was increased in SNP-treated cells but decreases when thalidomide, at 50 and 75 μg/ml, was used in combination (Figure 13). Similar effects of SNP and thalidomide were observed when cells were analyzed for filopodia and stress fibers formation (Figure 13 and 14).

### Thalidomide arrests SNP induced migration of ECs

**Figure 15:**
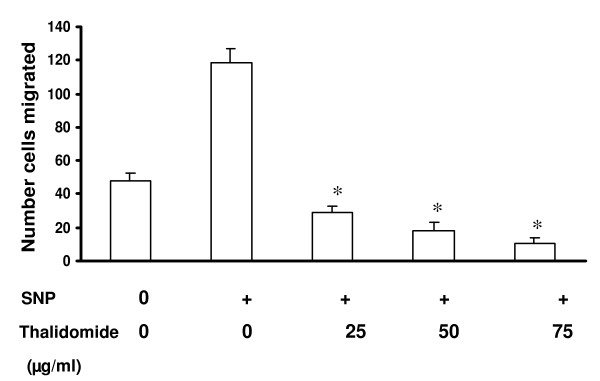
**Migration assay by using modified Boyden's chamber**: ECs were trypsinized and seeded on the upper chamber of Boyden's chamber, which is a twochamber system separated by a collagen coated 8-μm pore size polycarbonate membranes. Lower well was filled with DMEM. The chambers were then incubated at 37°C, 5% CO2 for 3 h. Cells were migrated across the membrane and stuck to the lower part of the membrane. After the incubation, the polycarbonate membrane was fixed and stained with propidium iodide, a fluorescent nuclear probe. Cell number was counted before and after experiments to quantify the proliferation status of the loaded cells in Boyden's chamber. * *P *< 0.05 compared to only SNP treated cells.

Boyden's chamber set up was employed to measure the collective migration pattern of ECs under SNP and SNP + thalidomide treatments. Analysis of Boyden's chamber experiments shows that all the concentrations of thalidomide arrested completely the SNP induced migration of ECs (Figure 15). Cell count remained same before and after the experiments, which further proves that thalidomide interferes with SNP mediated migration of ECs.

### Thalidomide reduces sub-population of the SNP treated cells with highermembrane actin polymerization pattern

**Figure 16:**
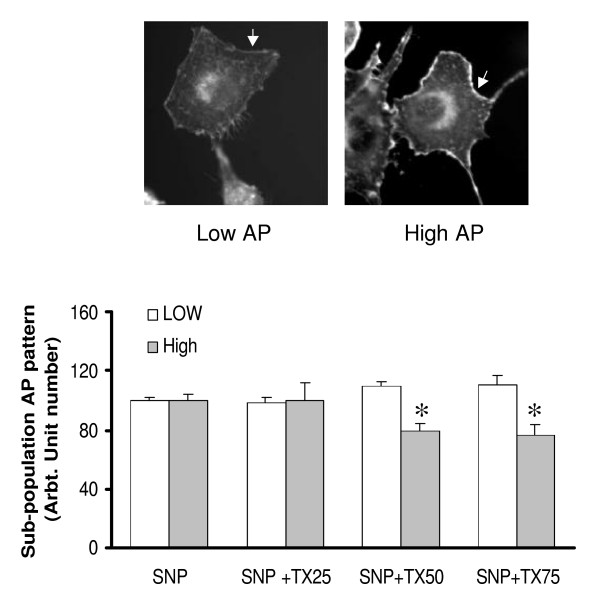
**Thalidomide reduces the sub-population of the SNP treated cells with higher membrane actin polymerization pattern: **Analysis of the images obtained from phalloidin experiments gives two sub-populations with varying fluorescent intensity of phalloidin 1) low intensity membrane pattern of actin polymerization described as Low AP and 2) Higher intensity of actin polymerization in the plasma membrane described as High AP (upper panel). SNP treatments of ECs caused an increase in the number of high AP cells. Further treatments of these cells with three concentrations of thalidomide (25, 50 and 75 μg/ml) reduced the number of high AP cells in a dose dependent manner. Particularly, 50 and 75 μg/ml groups significantly reduced the high AP population with a corresponding increase in low AP cells. SNP, Tx25, Tx50 and Tx75 denote 500 μmol SNP, 25, 50 and 75 μg/ml thalidomide respectively. The asterisks represent the level of significance. * *P *< 0.05 compared to only SNP treated cells.

Analysis of the images obtained from phalloidin experiments indicates that cells are divided into two sub-populations with low actin polymerization (low AP) and high actin polymerization (high AP) in relation to fluorescence intensity (Figure 16). SNP treatments of ECs caused an increase in the number of high AP cells, an effect that was reversed by thalidomide (25, 50 and 75 μg/ml) in a dose-dependent manner. The reduction of the high AP population was associated with a corresponding increase in low AP cells (figure 16).

## Discussion

In 1994, an ophthalmologist at Harvard Medical School discovered that thalidomide inhibits angiogenesis when tissues become deficient in oxygen [22]. The specific mechanism by which thalidomide inhibits angiogenesis is, as of yet, not known [23]. In our present work, we tested the anti-angiogenic potential of thalidomide by using egg yolk vascular bed. Results indicate that thalidomide blocks growth of the blood vessels at the periphery of the vascular bed (figure 1). We speculate that thalidomide affect the EC migration to attenuate angiogenesis at the cellular level. Thalidomide is also involved in the process of inflammation [24, 25, and 26]. Inflammation and cellular migration are two indispensable cellular mechanisms that complement each other [27]. Through experimentation in rabbits and in doing case studies on people being treated with thalidomide, researchers determined that thalidomide modulates the production of the inflammatory cytokine, TNF [23]. TNF-alpha is a cytokine produced by immune cells in the blood stream that acts as pro-angiogenic factor [28]. Thalidomide inhibits TNF-alpha by amplifying the degradation of messenger RNA (mRNA), and decreases the production of interleukin-12, which is involved in immunity responses, the stimulation of inflammation, and suppression of certain cytokines [29-31]. From these observations we delineate an association between cellular migration and thalidomide actions in ECs.

Our present work documents that thalidomide attenuates basal and NO-mediated EC migration in a dose-dependent manner (figure 3, 4, 9). Hence, in addition to NO signaling thalidomide appears to affect the migratory pattern of ECs by interfering other signaling pathway/s as well. Contrary to our assumption, single tube structure in the monolayer of ECs was resistant to thalidomide and SNP treatment in short-term experimental models (figure 10). We can speculate that the level of maturity of tubes is important for the effects of SNP and thalidomide as our results showed that nascent tubes were resistant to the effects of thalidomide (figure 10). Experiments in which phalloidin labeling of actin was performed indicate that thalidomide inhibits actin polymerization at the cell-cell interface (figure 5 and 6) and sensitizes cells to extend cell surface protrusions before completing a tube structure (upper panel, figure 10). Therefore, it is a plausible hypothesis that significant re-arrangements of actin cytoskeleton pattern during tube formation triggers membrane changes in ECs to protect the prima facie tube structures from the effects of excess growth factors and autacoids such as NO. This observation also supports the concept that thalidomide restricts blood vessel formation by inhibiting cellular proliferation and migration at the single cell level before they organize into a tube or ring like structure.

Dynamic rearrangement of the cytoskeleton is the key to migration of the cells [32]. Signaling pathways attributed to the migration of cells form a complex network of small GTPase-driven local networks that ultimately converge into the controlling nodes for actin polymerization pattern in the down-stream events [33]. Signaling pathways involving small GTPases and down stream activators such as ROCK, cdc42, MLCK tune the actin polymerization pattern in the cell membrane to define filopodia, a parallel arrangements of F-actins and lamellipodia, a diagonal arrangements of F-actins [21]. Dynamics of sub-cellular actin polymerization defines the cell surface extensions such as filopodia and lamellipodia structures, which are crucial to cellular migration [34]. With respect to actin polymerization, VEGF-treatment leads to rapid phosphorylation of actin, depolymerization factor cofilin, and its upstream regulator, LIM-kinase (LIMK). Pharmacological inhibitors of phosphoinositide-3 kinase (PI3-K) and the rho-activated kinase (ROCK) attenuate VEGF-induced LIMK phosphorylation that indicates a role for (PI3-K) and ROCK in the signaling pathways leading to regulation of LIMK activity [36]. We observed an altered phalloidin staining pattern, which represents the actin polymerization status of thalidomide treated ECs (figure 5 and 6). The number of lamellipodia was reduced significantly in thalidomide-treated cells while a strong pattern of central microfilament and membrane dense actin plates appeared in the cytosol and membrane respectively (figure 5). With respect to sub-cellular actin polymerization, a recent publication suggests that the sub-cellular localization of actin-related protein 2/3 (Arp2/3) complex, a prime actin polymerization nucleator, is crucial to the migration of the cells [36]. These observations prompt us to speculate that thalidomide signals to downstream effectors to impair migratory mechanisms of ECs by changing the actin polymerization pattern during the initial phase of angiogenesis by blocking individual cells to organize a tube like structure.

Does thalidomide cross-talk with NO downstream signaling pathway to interfere with cellular migration? To check the role of thalidomide in NO driven endothelial rearrangements we delivered NO via SNP prior to thalidomide treatments of the cells. We found that thalidomide neutralizes NO induced changes in the cytoskeleton of ECs (data not shown). This prima facie observation supports our hypothesis that thalidomide may cross talk with NO signaling pathway. Bioavailability and functions of NO are critically controlled by the redox status of the system [37]. Reports suggest that thalidomide enhances superoxide anion release from human polymorphonuclear and mononuclear leukocytes [38]. Therefore, relationship of NO and thalidomide with other oxygen species such as peroxynitrite, hydrogen peroxide, superoxide radicals and nitrite oxide would determine the nature of cross talk between thalidomide and NO down stream signaling. Recent reports claim that thalidomide exhibits NOS-inhibitory activity [39]. Although the results of our study support the thought of an interplay between thalidomide and NO downstream signaling pathway, it is possible that thalidomide exerts its anti-migratory effects, at least in part, by inhibiting NOS at the NO upstream events. The possible explanation is that inactivation of NOS by thalidomide is associated with the re-localization of NOS due to cytoskeletal rearrangements. Reorientation of actin-based infra-structure could place NOS in discrete membrane compartments or cytoplasmic regions causing insufficient access to other signaling proteins, which are responsible for NOS activation. There is an inverse relationship between the concentration of G-actin and eNOS expression in endothelial cells subjected to pharmacological alteration of their cytoskeleton [40]. The increase in eNOS activity caused by G-actin was much higher than that caused by F-actin [41]. However, our results document a higher degree of polymerization of actin in thalidomide-treated cells, which lead to expanded cell surface and perimeter as well (Figure 12). We speculate a possible mechanism of thalidomide mediated blocking of NO driven endothelial functions, which attributes, at least in part, to the thalidomide associated cytoskeleton rearrangement causing NOS inactivation. Hence, results of our present work strongly indicate that thalidomide blocks NO driven endothelial functions by interfering with the actin polymerization pattern of ECs. We deem that thalidomide attenuates NO driven endothelial functions, in part, in ECs by interplaying with the NO down stream signaling molecules.

## Conclusion

Present work evidences a novel cellular mechanism of thalidomide mediated endothelial dysfunction, which attributes to the altered pattern of EC migration. Further investigation into the cause of thalidomide-mediated disruption in NO-induced EC migration shows that thalidomide induces dose-dependent actin polymerization in EC in the early phase of angiogenesis, which might be the basic mechanism of the attenuation of angiogenesis. An extended study aiming at the dissection of biochemical signaling pathway, which will unravel the cross talk between thalidomide and NO down stream signaling would help to understand further the thalidomide actions in endothelium, an area of immense clinical importance.

## Abbreviations

AIDS – Acquired Immunodeficiency syndrome

Ang1 – Angiopoietin-1

AP – Actin Polymerization

Arp – Actin related protein

cGMP – Cyclic guanosine monophosphate

DEA-NONOate – 2-(N,N-Diethylamino)-diazenolate-2-oxide.diethylammonium salt

DMEM – Dulbecco's modified Eagle's medium,

DMSO – Dimethyl Sulfoxide,

EC – Endothelial cells,

ENL – erythema nodosum leprosum

ENOS – Endothelial Nitric oxide synthase

FBS – Fetal Bovine serum.

FDA – Food and Drug Administration

HSP90 – Heat shock Protein 90

IL-6 Interleukin-6

LPS – Lipopolysaccharide

MLCK – Myosin Light Chain Kinase

muTNF-alpha – Murine Tumor Necrosis Factor-alpha

NO – Nitric oxide,

NOS – nitric oxide synthase

PBS – Phosphate buffer Saline.

PI3-kinase – phosphatidylinositol 3-kinase

ROCK – Rho-associated kinase

SLE – Systemic lupus erythematosus

SNP – Sodium Nitroprusside,

Thalidomide-α-(N-phthalimido) glutarimide

TNF – Tumor Necrosis Factor

Tx – Thalidomide

VEGF – Vascular Endothelial Growth Factor

## Competing interests

The author(s) declare that they have no competing interests.

## Authors' contributions

KT and GK carried out the bright field and fluorescence microscopy of thalidomide effects on endothelial cells. MR analyzed cellular migration related data. I and S with KT standardized the experimental protocol for egg yolk angiogenesis assay. SC coordinated and helped interpreting the data. All authors read and approved the final manuscript.
